# Using a signal detection approach to understand the impacts of processing fluency and efficacy on accuracy in misinformation detection

**DOI:** 10.3389/fpsyg.2024.1417910

**Published:** 2024-09-16

**Authors:** Kara S. Fort, Hillary C. Shulman

**Affiliations:** School of Communication, The Ohio State University, Columbus, OH, United States

**Keywords:** misinformation, metacognition, information processing, experimentation, processing fluency, signal detection

## Abstract

This experiment (*N* = 1,019) examined how a state of processing fluency, induced through either an easy or difficult task (reading a simple vs. complex message or recalling few vs. many examples) impacted participants’ ability to subsequently detect misinformation. The results revealed that, as intended, easier tasks led to higher reports of processing fluency. In turn, increased processing fluency was positively associated with internal efficacy. Finally, internal efficacy was positively related to misinformation detection using a signal detection task. This work suggests that feelings of ease while processing information can promote confidence and a more discerning style of information processing. Given the proliferation of misinformation online, an understanding of how metacognitions – like processing fluency – can disrupt the tacit acceptance of information carries important democratic and normative implications.

## Introduction

There is a widespread and growing concern about the impacts of misperceptions and misinformation across a variety of contexts, but particularly online (Aslett et al., 2024; Loomba et al., 2021; McCarthy, 2021). Although there are different definitions of misinformation (including disinformation), here we use the term *misinformation* to mean information that is inaccurate, either by mistake or by design (Fallis, 2015). Guided by this definition, we are concerned with how misinformation, regardless of whether it was produced intentionally or unintentionally, can mislead people (Fallis, 2015). Recent experimental research has shown that exposure to misinformation about vaccines lowered vaccination intention by around six percentage points in both the United Kingdom and the United States (Loomba et al., 2021) and other research has linked increased belief in election conspiracy theories in the United States with eroding public confidence in democracy (Sanchez and Middlemass, 2022). To combat these effects, it is important to understand how individuals come to accept false information in the first place.

**Figure 1:**
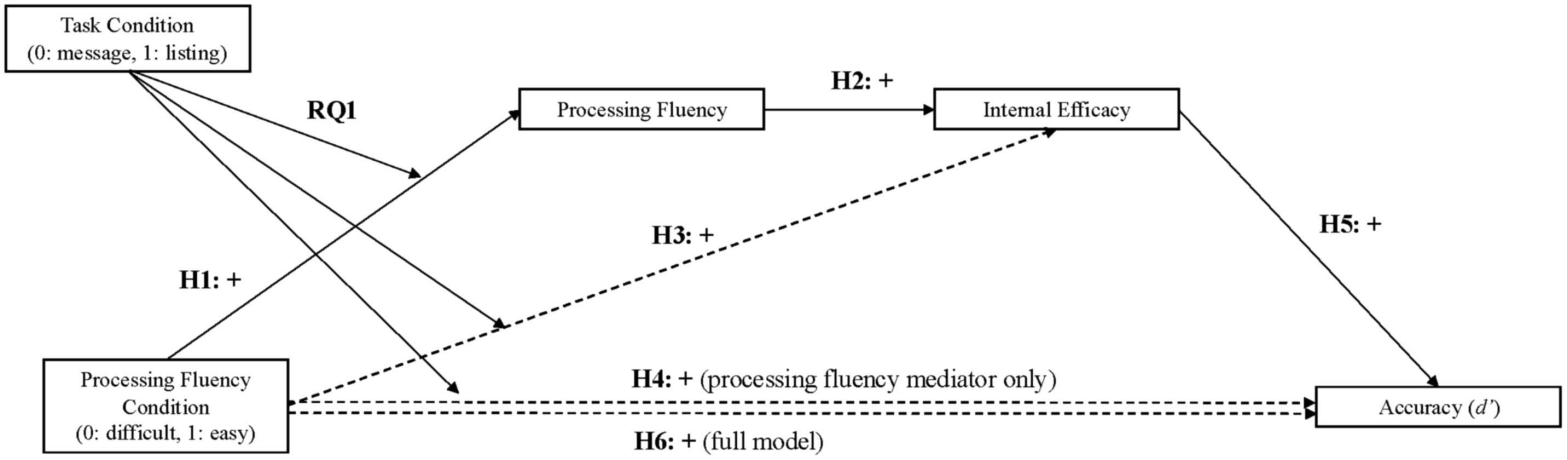
Hypothesized serial mediation model predicting accuracy. This figure illustrates the predicted relationships across the entire model under investigation. Notably, H1–H5 reflect path-by-path predictions that build towards the larger, and primary, hypothesis (H6) in this paper.

Through this experiment, we seek to understand how *metacognitions*, or thoughts about our thoughts (Petty et al., 2007) while processing information, impact the degree to which people scrutinize the validity of the information at hand. Currently, many of the existing approaches dedicated to understanding misinformation acceptance focus on the content and form of the messages themselves. This important work has shown that message features such as repetition (Bacon, 1979; Begg et al., 1992; Boehm, 1994; Hasher et al., 1977), belief or attitude congruence (Flynn et al., 2017; Priedols and Dimdins, 2023; Susmann and Wegener, 2023; Vegetti and Mancosu, 2020), the inclusion of non-probative photos (Newman et al., 2012; Newman and Zhang, 2021), and presenting true information prior to false information (Barchetti et al., 2022) all increase people’s receptivity to false information. Another strand of work in the space has focused on how individual differences, such as conservativism (Garrett and Bond, 2021), epistemological beliefs (Garrett and Weeks, 2017), and conspiratorial thinking (Bruder et al., 2013), among others function to promote misinformation acceptance as well. In this paper, we contribute to this discussion by seeking to understand how a person’s experiences while processing information, an important yet less studied component of information processing in this context (Petty et al., 2007; Schwarz, 2015) impacts peoples’ receptivity to the information being presented. To make these arguments, this paper is structured such that each hypothesis, in a sequential process, is explained, and builds towards our larger model which ultimately proposes that putting people in a heightened state of processing fluency (through experimental induction) will increase feelings of efficacy which, in turn, will improve people’s ability to detect misinformation (see Figure 1).

To make this case, our first section will explain how to evoke processing fluency using manipulations external to the message content to lay the foundation for hypothesis 1 (H1). The second section will advance how processing fluency should impact efficacy (H2) and potentially misinformation detection (H3). The final section then puts these ideas together by examining the relationship between processing fluency (H4) and efficacy (H5, H6) on misinformation detection. Thus, H6 offers a test of the complete model (Figure 1) and is the primary focus of this work.

### Feelings of ease while processing information

Metacognition is an area in social psychology (for reviews, Petty et al., 2007; Schwarz, 2015; Shulman and Bullock, 2019) that is interested in how thoughts about one’s thoughts impact judgments and evaluations. Although there are many types of metacognitions, in this paper we focus on *processing fluency,* which can be defined as the subjective feelings of ease or difficulty while processing new information (Schwarz, 2011). In other words, processing fluency reflects a person’s assessment of how easy, manageable, or familiar a message feels while processing (Schwarz, 2011). To offer an example of processing fluency, imagine the feelings one experiences after finishing an exam. Sometimes people walk out of an exam feeling positive and optimistic, because the exam felt easy to complete. Other times people feel immediately pessimistic about their performance because the exam felt difficult. Notably, in both scenarios, feelings of ease or difficulty led a person to make inferences about how well they performed on the exam. This is the power of processing fluency as a source of information. What is important to recognize, however, is that these feelings are not perfectly diagnostic of performance. In other words, sometimes these feelings are wrong, and we surprise ourselves (by doing much better or much worse than we thought). Importantly, however, these feelings are diagnostic often enough that people instinctively rely on these metacognitive feelings as a source of information. This is the first postulate of feelings-as-information theory (FIT, Schwarz, 2011), and the focus of this section: People attend to metacognitions as a source of information.

Schwarz (2006) and others (e.g., Miele and Molden, 2010; Oppenheimer, 2008) refer to the instinct to rely on metacognitive feelings, such as processing fluency, to make inferences about what these feelings mean in a particular context as *naïve theories*. Originally proposed by Heider (1958), naïve theories refer to the tendency of individuals, as social perceivers, to develop explanations to describe themselves, others, and the world around them (Wegener and Petty, 1998). These naïve theories influence individuals’ evaluation of their present subjective experiences and information. In this vein, research has found that more fluent, or easier, information experiences are interpreted positively via naïve theory (Schwarz, 2006). This is because easy processing *feels* good, so information processed easily must *be* good. Through this relationship, as feelings of ease become connected with the subject of one’s attention (i.e., the idea of embodied cognition, Schwarz and Lee, 2017), more positive evaluations of the subject at hand become likely. Guided by this postulate, it has been found that information processed fluently is more likely to be evaluated as true (Reber and Schwarz, 1999; Markowitz and Hancock, 2016; McGlone and Tofighbakhsh, 2000; Brennan and Williams, 1995), is perceived as more familiar (Tversky and Kahneman, 1973; Reber and Zupanek, 2002), and is evaluated as safer and less risky (Shulman et al., 2020; Song and Schwarz, 2009). All told, easy processing experiences tend to promote more message acceptance, because the positive feelings derived from one’s processing experience become attributed towards the information itself. The question we interrogate is whether this processing style can impact the detection of misinformation.

Before linking these ideas to misinformation, the necessary first step is to consider strategies that should effectively evoke a state of processing fluency or disfluency. One such strategy, recently studied within communication (see Shulman and Bullock, 2019), is message design. Specifically, the way in which information is presented, such as the use of infographics (Riggs et al., 2022) or the narrative form (Bullock et al., 2021), can positively impact processing fluency. Also, the language used in the conveyance of information, such as the use of semantically simpler words (Oppenheimer, 2006), the presence of more familiar or commonly used words (Shulman et al., 2022), or syntactically simple sentence structures (Tolochko et al., 2019), can all make the processing of information feel more fluent. Another factor that can impact fluency is message repetition. Recent research (So and Song, 2023) found that message repetition functions to make the message content feel easier to process due to familiarity. Processing fluency can also be manipulated via thought exercises. For instance, thought retrieval tasks that ask people to come up with many examples, as opposed to only a few examples, will elicit a more difficult and thus more negative processing experience (Schwarz et al., 1991). The reason for this is because processing fluency is associated with effort. Because recalling many examples is more effortful than recalling few examples, processing fluency has been found to decrease as more examples are required (see Alter and Oppenheimer, 2009). Thus, taken together, the presentation of information, whether through message design or repetition, or engaging in a task that may be cognitively easy or difficult, can impact feelings of processing fluency.

Here, to understand whether a state of processing (dis)fluency can eventually impact the detection of misinformation, our experiment requires that we successfully manipulate feelings of ease or difficulty *before* exposing people to misinformation. As such, we include this manipulation check in our larger model so that – statistically – this cognitive state performs as a mediator that allows us to observe the empirical relationship between a state of processing fluency, efficacy, and eventually misinformation detection (see Hayes, 2009; O’Keefe, 2003, for more information about this analytical strategy). In this way, we can understand how metacognition, independent of misinformation type or content, can impact how critically people evaluate subsequent information. Guided by our discussion on the different approaches to instantiating a state of fluency (for a review of manipulations: Alter and Oppenheimer, 2009), we chose to manipulate processing fluency in two ways. Specifically, we chose a language complexity manipulation (Shulman and Sweitzer, 2018) and a thought retrieval task (Schwarz et al., 1991). The language complexity manipulation will present participants with a message about technology regulation (a topic generally related to misinformation online). In the difficult condition, idiosyncratic and highly pedantic words will be used to explain the regulation (e.g., falsify). In the easy version, these words will be replaced with simpler, and more common alternatives (e.g., reject). The thought retrieval task will ask participants to list either two leaders of technology companies (easy condition) or eight leaders (difficult condition). Importantly, both of these approaches are consistent with previous processing fluency inductions (Alter and Oppenheimer, 2009). That said, although we use two manipulations, to better understand the generalizability and potential nuances of these methodological decisions (see Slater and Gleason, 2012, for a discussion of the benefits of multiple manipulations), we predict the same relationship with processing fluency. Namely, we hypothesize:

*H1*: Reports of processing fluency will be higher in the easy processing fluency conditions (simple language, fewer examples) than the difficult processing fluency conditions (complex language, more examples).

Although our predictions will be the same regardless of processing fluency manipulation, given that we are taking two approaches to manipulating processing fluency, we can assess whether one manipulation of processing fluency is more successful than the other in this context. Research has suggested that all manipulations of processing fluency produce similar results, such that more fluent messages are perceived more positively than less fluent messages (Alter and Oppenheimer, 2009). Schwarz (2004) even went as far as saying that “theoretically, any…variable that increases processing fluency should have the same effect” (p. 338). We take this opportunity to test these claims and compare the effects of two processing fluency manipulations in the same study. By doing so, we can ask the following research question:

RQ1: Will there be an interaction effect between task condition (language, listing) and processing fluency condition (easy, difficult) on self-reports of processing fluency?

### Processing fluency and self-perceptions

If a state of processing fluency is successfully induced (H1), then the question becomes what effect this will have on cognitions and outcomes of interest. Naïve theories refer to the notion that one’s feelings while processing information can be diagnostic of either the quality of the information itself (as positive or negative, familiar or unfamiliar, of high or low quality), or one’s relationship to this information (Petty et al., 2007). Here we focus on this latter association. Specifically, processing fluency has been shown to be diagnostic about the state of one’s knowledge (Reber and Schwarz, 1999). This idea was foreshadowed previously with the example about feelings after taking an exam. If the exam felt easy, people assume they did well (and thus are knowledgeable about the subject). If the test felt difficult, people assume that they must not know much about the material. This idea reflects the notion that people make inferences about the state of their knowledge from experiences of ease while processing information. This section discusses this naïve theory and its relevance to misinformation detection.

When processing information feels easy, people naively assume that processing feels easy because they are knowledgeable about, or familiar with, the information at hand. To illustrate this idea, in an experiment by Shulman et al. (2020), participants were exposed to a science message that either included complex words, such as science jargon, or simpler words. They found the use of simpler words increased processing fluency. Interestingly, participants who reported higher levels of fluency also reported that they were more of a “science person” and that being a science person was “important to them.” In other words, an easier experience impacted their self-schema (Markus, 1977). Other work in different domains such as politics has similarly found that an easier experience (evoked through language simplicity) increased one’s internal political efficacy (Shulman and Sweitzer, 2018), and perceptions of their own political knowledge (Bishop et al., 1984). These relationships were also obtained in the context of health information, where participants reported higher health/risk internal efficacy alongside gains in processing fluency (Kim and Jang, 2018; Okuhara et al., 2020; Shulman et al., 2022). These ideas substantiate Petty et al.’s (2002) self-validation hypothesis, which asserts that easy experiences while processing information can enhance one’s self-confidence and, in turn, lead to more focused attention to the subject matter. Thus, in sum, research has shown that an easy experience leads people to (perhaps naively) assume that they are more knowledgeable, or efficacious, about the subject at hand. To capture these ideas, we use Niemi et al.’s (1991) construct of *internal efficacy* in this experiment. Internal efficacy can be defined as a person’s belief that they are able to effectively understand and engage with information about current events (our chosen research topic, given we are interested in general forms of misinformation online). With this definition in place, we hypothesize about each of these relationships below:

*H2*: Self-reports of processing fluency will be positively associated with internal efficacy.

*H3*: There will be a positive indirect relationship between processing fluency condition (easy or difficult) and internal efficacy through the mediator of self-reported processing fluency.

### Putting it all together: evaluating the accuracy of information

The ultimate goal of this investigation is to understand whether a state of fluency improves or degrades misinformation identification. In this experiment, to test people’s accuracy at detecting misinformation, we use a signal detection task as our outcome of interest (Stanislaw and Todorov, 1999). This task, described in more detail in the next section, measures two dimensions related to accuracy. The first, referred to here as *accuracy*, can be described as people’s ability to accurately distinguish true information from false information. The second dimension of a signal detection task is called *bias*. Bias measures peoples’ propensity to systematically respond “true” (a liberal bias) or “false” (a conservative bias). Thus, this task allows us to test accuracy alongside any influential response biases.

With these ideas in place, we have endeavored to provide a foundation that links a state of fluency (induced by a manipulation external to the message) to increases in efficacy. With these relationships articulated, we can now consider whether an easier fluency experience engenders positive feelings (Schwarz, 2006), and tacit message acceptance (Shulman and Bullock, 2019) or whether a more fluent experience engenders more careful message processing. The argument for the latter is that if fluency impacts self-perceptions and builds internal efficacy, it is also possible that this efficacy produces more systematic thought, and thus more accurate misinformation detection, as a result. The question of whether a state of fluency produces more heuristic processing (i.e., tacit acceptance of information, McGlone and Tofighbakhsh, 2000; Reber and Schwarz, 1999) or more systematic processing (i.e., more discernment) is a rich debate in the field on metacognition (see Petty et al., 2002; Petty et al., 2007; Schwarz and Lee, 2017; Shulman and Bullock, 2020; Shulman et al., 2022). Because the detection of misinformation requires a degree of vigilance, or deliberate thought to detect, we strive to address this theoretical debate within a timely and important context. Specifically, big picture, if a state of fluency makes people more prone to misinformation acceptance, than this evidence suggests fluency produces a more heuristic processing state. Alternatively, if fluency produces more accuracy in detecting misinformation, than a more systematic processing state is implied. We outline these ideas and the methodological decisions made to test these claims below.

First, it is important that we state that we chose to put people in a state of fluency (or disfluency) before exposure to the misinformation detection task. In other words, our processing fluency manipulations did not occur alongside, or within, the misinformation messages themselves. Instead, the manipulation occurred before the misinformation task was presented. We chose this approach because prior work has shown that *information context collapse*, or the tendency for social media sites to sort content by recency or popularity, rather than topic, that results in frequent changes in topic between posts (Pearson and Cappella, 2024), is associated with inattentive processing and source blindness (Pearson, 2021). Information context collapse occurs due to the simultaneous presentation of different types of content. For example, users on Facebook encounter current event, news content, and advertisements displayed alongside personal content without clear stylistic differences in the same newsfeed. This range of topics, without clear delineations between topics, can produce more passive information consumption and a general lack of content absorption (Baughan et al., 2022). This would suggest that individuals do not spend much time engaging with content while scrolling. In order to capture this state of mind, we are interested in the general impact of fluency experiences on subsequent message processing across multiple pieces of information. This method is more in-line with actual experiences while consuming a wide range of information online. Moreover, we posit that a fluent, or easy processing experience is likely to feel a lot like scrolling through a newsfeed online; passive and relatively effortless. Extrapolating this claim to the current context, we are interested in assessing how a state of metacognitive ease might associate with misinformation acceptance. Together, taking an ecological approach to studying the effects of metacognition on message acceptance is also practically important given that 86% of US adults report sometimes getting news from a smartphone, computer, or tablet (Pew Research Center, 2023).

If processing fluency eventually impacts evaluations of validity, or accurate misinformation detection, it likely does so because fluency evokes a particular processing style: either heuristic or systematic. In the context of misinformation, we assume that processing style should impact misinformation detection such that a more systematic style should produce better detection than a heuristic style. To offer some background, dual-process models of information processing posit that there are two processing routes. One route typically engages in less effortful heuristic and automatic processing while the other is effortful and engages in deliberation and analytical processing. There are numerous dual-process theories (e.g., Heuristic-Systematic Model, Eagly and Chaiken, 1993; System I and System II, Kahneman, 2011; Elaboration Likelihood Model, Petty and Cacioppo, 1986), however for clarity we will simply refer to processing as either heuristic or systematic.

There is much evidence to suggest that processing fluency is associated with increased heuristic processing. For example, when given a cognitive reflection task, which is a measure of heuristic processing, participants engaged in more systematic processing when shown questions in a more difficult to read font, and more heuristic processing when shown questions in easy-to-read fonts (Alter et al., 2007). Similarly, Song and Schwarz (2008a) asked participants how many animals *Moses* took on the ark, when in fact it was *Noah*. Participants given the question in a more difficult to read font were more likely to notice the error than those given an easy-to-read font. Thus, there is some evidence to suggest that processing fluency leads to the heuristic processing of subsequent information.

That said, there is also evidence that processing fluency might be associated with higher levels of systematic processing. For example, Domgaard and Park (2021) found that participants that were given a health infographic, which was more fluent than a news story without an infographic, were better able to integrate the information and identify false health news than participants who viewed the news and control (no stimuli) conditions. Additionally, participants in the infographic condition displayed lower trust in false news and had higher confidence in their judgments than participants in the control condition. This suggests that fluency may allow for more systematic processing of information via the reduction of cognitive load and an increase in confidence.

To synthesize these ideas, the goal of this work is to understand whether processing fluency, a state of mind likely to be experienced while people browse information online, impacts misinformation detection. We see two possibilities derived from the dual-process theories outlined above. The first possibility draws from FIT (Schwarz, 2011) and the naïve theory that suggests if a person is experiencing a state of fluency, they will be more likely to accept the information they are processing and interpret this information as true (Reber and Schwarz, 1999; Markowitz and Hancock, 2016; McGlone and Tofighbakhsh, 2000; Brennan and Williams, 1995). These ideas are in line with a heuristic processing path and culminate around the expectation that a state of fluency should lead people to be *worse* at detecting misinformation.

*H4*: There will be a negative indirect relationship between fluency condition (easy, difficult) and accuracy in accuracy ratings, mediated by self-reported processing fluency.

Although processing fluency may lead to tacit information acceptance (regardless of veracity), a read of the metacognition literature informs a second plausible interpretation as well. Instead of necessarily promoting tacit information acceptance, a state of fluency could enhance people’s self-perceptions of their ability to engage with information about current events (i.e., self-validation hypothesis, see Petty et al., 2002). In other words, an easy experience should build perceptions of one’s confidence or internal efficacy. There is a precedent for this relationship in the theorizing within this literature (Petty et al., 2002; Petty et al., 2007; Tormala et al., 2002), and empirical evidence that supports this relationship (Shulman et al., 2020). This is because if information feels easy to process, people infer that they must be familiar with and knowledgeable about the information at hand. Additionally, research has shown that when processing feels easier, individuals feel more efficacious and are consequently more motivated to complete a proceeding task than they would be if processing was difficult (Kim and Jang, 2018; Song and Schwarz, 2008b). We extend these ideas here by testing whether perceptual gains in internal efficacy are associated with actual gains in a person’s ability to detect misinformation. In this experiment, we offer a preliminary test of this idea by examining whether processing fluency improves misinformation detection, through internal efficacy. This finding would provide support for the idea that processing fluency can catalyze more systematic processing for domain specific information, driven by enhancements to one’s perceptions of their internal efficacy.

*H5*: Internal efficacy will be positively associated with accuracy in veracity ratings.

In addition to these relationships, as stated in the introduction, it becomes important to test these relationships while accounting for individual differences that have been found to significantly impact misinformation detection. These individual differences include epistemic beliefs (Garrett and Weeks, 2017), conspiracy mentalities (Bruder et al., 2013), and political ideology (Garrett and Bond, 2021). Specifically, research in this vein has shown that individuals who believe that truth is politically constructed (Garrett and Weeks, 2017), have higher levels of conspiracist ideation (Bruder et al., 2013), and are politically conservative (in the US; Garrett and Bond, 2021) are more likely to believe false information. In contrast, individuals who report a reliance on external, validated evidence in their decision making are less susceptible to misinformation (Garrett and Weeks, 2017). By accounting for these traits, we can examine whether a cognitive state of fluency, and subsequent impacts on efficacy, can affect misinformation detection in ways above and beyond these well-documented individual differences.

With this foundation in place, we are now able to present a full serial model that postulates how a state of processing fluency might impact the detection of misinformation. In addition to explaining the cognitions that may underlie misinformation detection, we also resolve to make this case while accounting for individual differences known to enhance (or degrade) misinformation acceptance:

*H6*: There will be a positive indirect effect of the fluency condition (easy vs. difficult) on accuracy, through the serial mediators of reported processing fluency and internal efficacy.

## Method

### Participants

Participants in this online experiment were recruited via CloudResearch’s MTurk Toolkit (*N* = 1,019). This sample was 52.40% male, 44.2% female, 1% non-binary, with 1.4% reporting that they preferred not to answer. The sample ranged in age from 19 to 79 (*M* = 43.17, *SD* = 12.39). Additionally, 80.36% of the sample identified as white, 9.17% as Black or African American, 1.70% as American Indian or Alaska Native, 7.68% as Asian, and 4.79% as Hispanic (participants were allowed to identify as more than one race). To be eligible to participate, participants had to be at least 18 years old. To improve data quality, participants had to pass a CAPTCHA and had to have participated in at least 500 HITS (Human Intelligence Tasks) with a completion rate of at least 95%. Those eligible to participate were compensated $2.00 for completing the survey. The survey took an average of 12.61 min to complete (*SD* = 7.10, *Median* = 10.88).

### Experimental design and procedure

Participants were randomly assigned to experimental conditions using Qualtrics software in a 2 (processing fluency condition: easy versus difficult) × 2 (task condition: message versus listing) between-subjects design. All participants were asked to read either an article about technology regulation (message condition) or complete a thought listing task about technology leaders. We chose this topic because it is loosely related to perceiving information online, which was the ostensible purpose of this study (IRB approval #E2024E0184). These stimuli were held on screen for 8 s to encourage completion. Following the stimuli, questions about processing fluency were presented. All participants were then shown 20 fact-based statements (12 false, 8 true, described below), in random order, that were said to have circulated on social media. For each statement, participants were asked whether the statement was true or false. Then, questions on internal efficacy, epistemic beliefs, conspiratorial mentality, political ideology, and demographics were presented. After demographics, participants were shown a debriefing message which clarified that the statements were not necessarily from social media and corrected any misinformation they were shown.

Before moving on to the stimuli and measures, we do want to offer one note about the ordering of our variables. Although testing the impact of processing fluency on misinformation detection through internal efficacy is intriguing and theoretically important, we note one complexity for observing this relationship. Specifically, naïve theories operate when attention is not drawn toward their influence (i.e., incidental rather than integral, Schwarz and Clore, 2013). Thus, directly asking participants about their internal efficacy before a misinformation task could prime participants to think about their performance in ways that a processing fluency manipulation would not. Alternatively, asking participants about their efficacy *after* a detection task creates the possibility that the task influenced these self-reports. Although there was no obvious solution to this issue, given the strong theoretical precedent between processing fluency and internal efficacy (Petty et al., 2007; Shulman and Bullock, 2019; Schwarz, 2015), we opted to include the measure of internal efficacy after participants completed the misinformation task, not before as our model would indicate. We address this issue in the discussion section but wanted to mention this point about the procedure for transparency.

### Stimuli

Because we included two different processing fluency manipulations, we had two versions of the stimuli, a message version and a retrieval task version (each with an easy and difficult version). The first manipulation type, termed the message condition, presented a manipulated version of an article about technology regulations from The Hill (Klar, 2023). The difficult version of the message stimuli was most similar to the original article, with a few words replaced to increase the potency of this manipulation (Flesch–Kincaid Grade Level: 15.1)^1^. In the easy condition difficult words (e.g., falsify) were swapped with their simpler versions (e.g., reject) using a thesaurus (Flesch–Kincaid Grade Level: 8.8). This is the same approach used in prior research on language difficulty (Bullock et al., 2019; Shulman et al., 2020; Shulman and Bullock, 2020). The second manipulation type, termed the listing condition, asked participants to list either two technology leaders in the easy condition or eight technology leaders in the difficult condition. Similar manipulations have been used before and are referred to as “retrieval ease” tasks (Schwarz et al., 1991; Schwarz and Schuman, 1997). Complete versions of the stimuli are available in the Supplementary materials.

### Measures

A full list of measures is available in the Supplementary materials as well as a correlation matrix. All items were measured on a 1 to 7 scale in which higher scores reflect a stronger agreement with the concept being measured, unless otherwise noted. Descriptive statistics for the measures by the experimental conditions are presented in Table 1.

**Table 1:** Descriptive statistics of the outcome variables by experimental condition.

Condition	*n*	Processing fluency	Internal efficacy	Accuracy (*d’*)	Bias (ln[β])
Overall	1,019	4.29 (1.55)	4.81 (1.26)	0.98 (0.79)	−0.27 (0.69)
Easy conditions				
Message	260	4.37 (1.32)	4.76 (1.20)	1.01 (0.70)	−0.22 (0.60)
Listing	255	5.34 (1.34)	4.70 (1.33)	0.95 (0.83)	−0.29 (0.70)
Total	515	4.85 (1.42)	4.73 (1.27)	0.98 (0.77)	−0.26 (0.65)
Difficult conditions				
Message	259	3.83 (1.37)	4.80 (1.30)	1.02 (0.81)	−0.34 (0.76)
Listing	245	3.58 (1.55)	5.00 (1.15)	0.96 (0.80)	−0.23 (0.72)
Total	504	3.71 (1.46)	4.89 (1.24)	0.99 (0.80)	−0.29 (0.74)

These numbers reflect descriptive conditional means and thus do not include the impact of the influential covariates (epistemic beliefs, ideology, conspiracy mentality) used in hypothesis testing.

### Processing fluency

After exposure to the fluency manipulation, participants responded to a six-item measure assessing their processing fluency (Shulman and Bullock, 2020). Participants in the message version of the task received the standard version of the scale, while participants in the listing conditions responded to an adapted version of the scale that referred to a “task” instead of a “message.” The six items were averaged to create a single processing fluency measure (α_Message_ = 0.89, α_Listing_ = 0.92; see Table 1). An example item includes, “It was easy for me to recall the information.”

### Misinformation task

To assess participants’ ability to accurately assess the veracity of information, 20 fact-based statements were shown after the processing fluency scales. All participants saw the same statements, in random order. The statements were chosen to provide a variety of contexts and lengths (see Table 2 for a full list of statements). Consistent with prior research (e.g., Newman, 2013), we assessed a range of topics with these statements including conspiracy theories, trivia statements, celebrities, politics, and health and technology. For all topics (other than conspiracy theories because these were all false), two statements were true and two were false. Thus, there were 12 false statements and 8 true statements. The conspiracy theory (see Garrett and Weeks, 2017) and the trivia and celebrity (see Newman, 2013) statements had been used in prior research. The political statements were real political claims that had been fact checked by organizations like PolitiFact and https://www.factcheck.org/ and were balanced in terms of political lean. Finally, the health and technology statements were real claims that had been fact-checked by https://www.factcheck.org/. After viewing each statement, participants responded to one item that asked whether the statement they read was true or false.

**Table 2:** Misinformation statements.

	True statements	False statements
Conspiracy theories		The assassination of John F. Kennedy was not committed by the lone gunman Lee Harvey Oswald but was rather a detailed organized conspiracy to kill the President. The assassination of Martin Luther King Jr. was the result of an organized conspiracy by U.S. government agencies such as the CIA and FBI. Princess Diana’s death was not an accident by rather an organized assassination by members of the British royal family who disliked her. A powerful and secretive group known as the New World Order are planning to eventually rule the world though an autonomous world government which would replace sovereign governments.
Trivia	Neil Armstrong was the first person on the moon. A one-lens eye piece is called a monocle.	An ostrich is a pink colored bird that stands on one leg. Galileo discovered gravity.
Celebrities	Stephen King is alive. Nina Simone is dead.	Joni Mitchell is dead. Dr. Seuss is alive.
Politics	Donald Trump deported less people than Barack Obama did during his presidency. Postpartum Medicaid coverage expanded from three states to 43 states because of the Biden administration.	Republican presidential candidate Nikki Haley supports a 23% national sales tax. None of the classified documents found in President Biden’s possession were highly classified.
Health and Technology	Electric vehicles contribute fewer emissions that gasoline-powered cars over their lifetimes. There are no proven health risks for the general population from consuming the artificial sweetener aspartame.	Thermography is an effective and FDA approved alternative to mammograms. Diagnoses of HIV in the U.S. military have increased 500% since the COVID-19 vaccine was mandated for service members.

Fact checking materials for the politics and health and technology topics are available in the Supplementary materials.

### Calculating accuracy via signal detection

We took a signal detection task approach (Stanislaw and Todorov, 1999) to calculate participant’s accuracy in detecting misinformation. A signal detection task is designed to assess a person’s ability to discriminate between hits and foils. In a recent paper applying signal detection to misinformation (Batailler et al., 2022), this test was used to identify people’s ability, termed *accuracy*, to discern true information (Hits, true positives) from false information or misinformation (Foils, true negatives). Instances where participants incorrectly stated a piece of information was true when it is not (false positive) or was not true when it is (false negative) can be characterized as noise. The test of accuracy used here is *d’* which can be interpreted as the distance (in standard deviation units) between the hit distribution and the foil distribution. Higher values indicate better accuracy.

In addition to accuracy, signal detection tasks also include a measure of bias, which is a calculation of a participant’s propensity to respond yes (i.e., “yes” this information is factual, a liberal bias) versus no (i.e., “no” this information is not factual, a conservative bias). Although not always the case, a conservative bias tends to be associated with more accurate deception detection (Levine, 2019). With this measure of bias, a truth bias can be interpreted as more negative scores (the natural log version of Beta, see Stanislaw and Todorov, 1999). More positive values indicate more of a no (false) bias. Thus, for our hypotheses that examine the veracity of misinformation, we assessed performance accuracy with both (*d’*) and bias (β).

We also note that there are two statistical assumptions in the calculations of these measures, normality and equal variances. Although, according to Stanislaw and Todorov (1999) there is no straightforward way to test whether these assumptions are met for yes/no tasks, we want to note that our measures for *d’* and β were significantly correlated (*r* = −0.606, *p* < 0.001), which Stanislaw and Todorov (1999) state is a sign that one or both of the statistical assumptions were violated.

### Internal efficacy

Internal efficacy was measured on a four-item scale. The measures were adapted from an internal political efficacy scale (Niemi et al., 1991), wherein references to politics, government, or political issues were replaced with either current events or current issues. The four items were averaged together to create a single measure of internal efficacy (*M* = 4.81, *SD* = 1.26, *α* = 0.84). An example item includes, “I feel that I have a pretty good understanding of the important current issues facing our country.”

### Individual differences

Prior work on misinformation has found that individual differences are often associated with misinformation acceptance or rejection (Garrett and Bond, 2021; Garrett and Weeks, 2017). As such, we include these measures here to examine whether processing fluency impacts misinformation detection above and beyond these individual differences.^2^ These covariates include epistemic beliefs, conspiratorial mentality, and political ideology. Epistemic beliefs were measured in a series of three subscales: faith in intuition for facts, need for evidence, and truth is political (Garrett and Weeks, 2017). All three subscales were measured with four items on a 1 (strongly disagree) to 5 (strongly agree) scale. The faith in intuition for facts scale measures an individual’s tendency to trust their gut. An example item includes, “I trust my initial feelings about the facts”; the four-items of this scale were averaged together to create a single measure of faith in intuition for facts (*M* = 3.18, *SD* = 0.92, *α* = 0.86). The need for evidence scale measures an individual’s tendency to rely on externally validated evidence (“A hunch needs to be confirmed with data”); the four items of this scale were averaged together to create a single measure of need for evidence (*M* = 4.29, *SD* = 0.71, *α* = 0.81). Truth is political measures an individual’s tendency to believe that fact cannot be separated from social and political processes (“Facts are dictated by those in power”); the four items of this scale were averaged together to create a single measure truth is political (*M* = 2.69, *SD* = 1.10, *α* = 0.85). Conspiracy mentality was measured on a five-item scale (Bruder et al., 2013). An example item includes, “I think that there are secret organizations that greatly influence political decisions.” The five items were averaged together to create a single measure of conspiracy mentality (*M* = 4.55, *SD* = 1.40, *α* = 0.87). Political ideology was measured on a 1 (very liberal) to 7 (very conservative) scale (*M* = 3.51, *SD* = 1.86).

## Results

**Figure 2:**
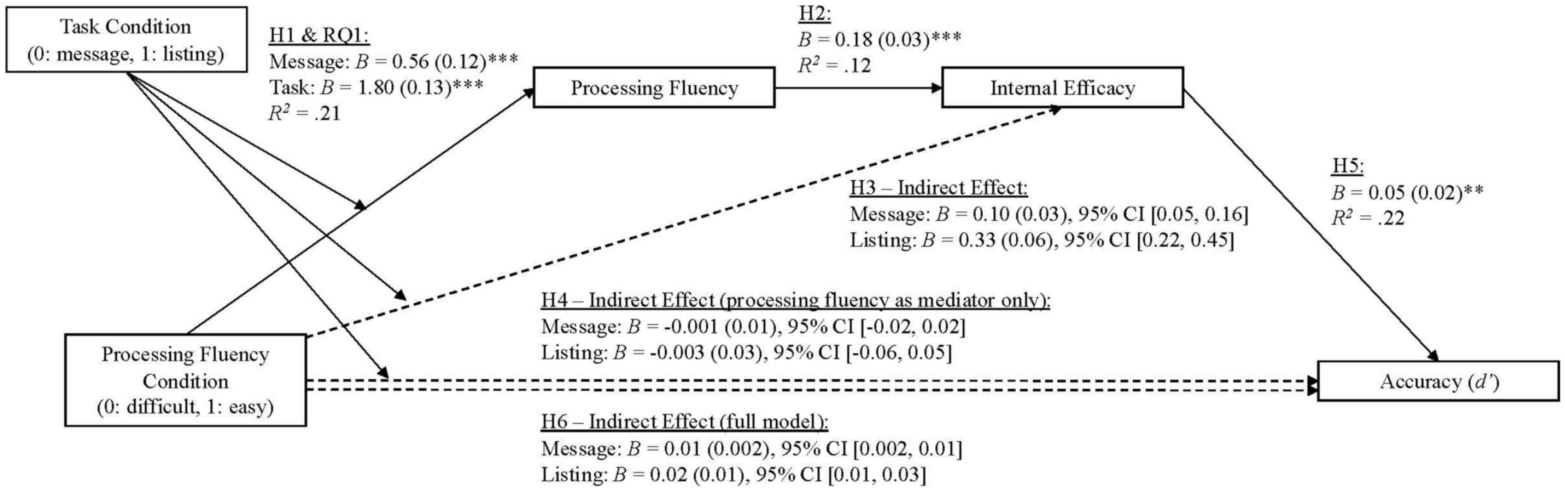
Serial mediation model predicting accuracy. Standard errors are shown in parentheses. Index refers to the index of moderated mediation. Participants’ epistemic beliefs, conspiracy mentality, and political ideology were included as covariates in the model. For all analyses, mediation is supported if the 95% confidence interval does not include zero. **p < 0.01, ***p < 0.001.

Given that all study hypotheses build towards the larger model depicted in Figure 1, we opted to run this larger model first, and then report upon each path pertinent to the hypothesis or research question being tested. In this way, all results are statistically consistent with one another (with the exception of H3 as this model had to be run separately because this model included a different dependent variable). The primary analysis was run with Hayes (2013) macro PROCESS Model 85 with the following parameters: 95% bias-corrected bootstrap confidence intervals based on 10,000 resamples with participant’s conspiracy mentality, political ideology, and epistemic beliefs included as covariates. This model is visualized in Figure 1 and the full set of results are available in Tables 3, 4. Hypothesis relevant results are visualized in Figures 2, 4.

**Table 3:** Serial mediation results predicting accuracy.

Predictors	Path 1 *B* (*SE*)	Path 2 *B* (*SE*)	Path 3 *B* (*SE*)
Intercept	2.88 (0.43)***	1.23 (0.37)***	1.05 (0.22)***
Processing fluency condition	0.56 (0.12)***	−0.10 (0.11)	−0.03 (0.06)
Task condition	–0.25 (0.13)*	0.24 (0.11)*	−0.09 (0.06)
PF × Task conditions	1.24 (0.18)***	−0.50 (0.15)**	0.06 (0.09)
Processing fluency		0.18 (0.03)***	−0.002 (0.02)
Internal efficacy			0.05 (0.02)**
Faith in intuition for facts	0.06 (0.05)	0.26 (0.05)***	−0.09 (0.03)***
Need for evidence	0.26 (0.07)***	0.38 (0.06)***	0.20 (0.04)***
Truth is political	–0.09 (0.05)	0.08 (0.04)	−0.06 (0.02)*
Conspiracy mentality	–0.02 (0.04)	−0.01 (0.03)	−0.12 (0.02)***
Political ideology	–0.01 (0.03)	0.07 (0.02)**	−0.04 (0.01)***
*F*	33.43***	14.86***	28.41***
*R^2^*	0.21	0.12	0.22

Path 1 indicates the unstandardized relationship between processing fluency condition (0: difficult, 1: easy) and self-reported processing fluency. Path 2 indicates the unstandardized relationship between self-reported processing fluency and internal efficacy. Path 3 indicates the unstandardized relationship between internal efficacy and accuracy (*d*’). *p < 0.05, **p < 0.01, ***p < 0.001.

**Figure 3:**
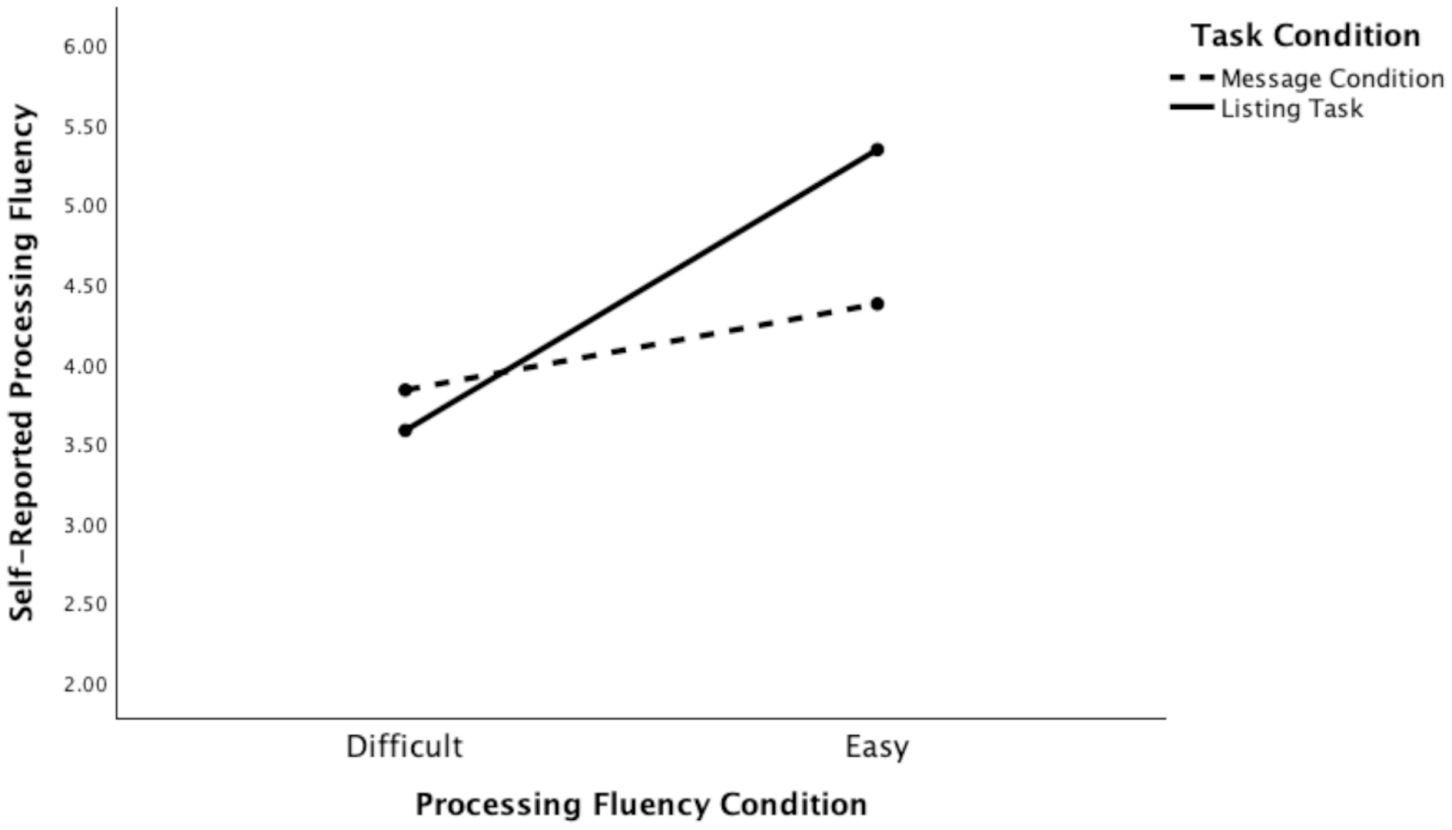
The effect of the processing fluency manipulations on self-reported fluency. This figure illustrates the effectiveness of the two experimental manipulations (H1) and their interaction effect (RQ1).

To begin, H1 and RQ1 concerned the impact of the task manipulations on processing fluency. These analyses can be found in the first path of our larger model. This analysis revealed that this part of the model was significant, *F*(8, 991) = 33.43, *p* < 0.001, *R*^2^ = 0.21. In support of H1, participants assigned to the easy processing fluency conditions reported significantly higher processing fluency scores than those in the difficult conditions (*B* = 0.56, *SE* = 0.12, *t* = 4.54, *p* < 0.001), even when accounting for all covariates. This analysis also revealed that there was a significant interaction (RQ1) between the task and processing fluency conditions, *B* = 1.24, *SE* = 0.18, *t* = 7.09, *p* < 0.001. This interaction effect is visualized in Figure 3 (see also Table 3, path 1) and suggests that the listing task was a stronger processing fluency manipulation than the message task. Taken together, these results provide support for H1 because the task manipulations both operated as intended. That said, given that the two task conditions were differentially effective, task condition was included as a moderator throughout subsequent analyses.

Hypothesis two predicted that processing fluency would be positively associated with internal efficacy. Overall, this portion of the model was significant, *F*(9, 990) = 14.86, *p* < 0.001, *R^2^* = 0.12. Consistent with H2, processing fluency was a positive predictor of internal efficacy, *B* = 0.18, *SE* = 0.03, *t* = 6.73, *p* < 0.001 (Table 3, Path 2). Thus, H2 was supported.

Hypothesis three predicted that there would be a positive indirect relationship between processing fluency condition and internal efficacy via the mediator of processing fluency. To test this hypothesis, Hayes’s (2013) PROCESS Model 8 was ran (95% bias-corrected bootstrap confidence intervals based on 10,000 resamples with conspiracy mentality, political ideology, and epistemic beliefs included as covariates). Consistent with H3, positive, non-zero indirect effects were found at both levels of the moderator (task condition) – message conditions, *B* = 0.10, *SE* = 0.03, 95% CI [0.05, 0.16]; listing conditions, *B* = 0.33, *SE* = 0.06, 95% CI [0.22, 0.45], *R*^2^ = 0.12; Index of moderated mediation (Index) *B* = 0.23, *SE* = 0.05, 95% CI [0.14, 0.33] (see Table 3 Paths 1 and 2 for full results). Taken together, these results suggest that participants in the easy conditions (across both tasks) reported higher levels of internal efficacy, through the mediator of processing fluency. Thus, this hypothesis was supported.

Hypothesis four predicted that participants in the easy conditions would be less accurate at detecting misinformation, via the mediator of processing fluency. The results indicate that the indirect effect was not distinguishable from zero at either level of the moderator (task condition) – message conditions, *B* = −0.001, *SE* = 0.01, 95% CI [−0.02, 0.02]; listing conditions, *B* = −0.003, *SE* = 0.03, 95% CI [−0.06, 0.05], *R*^2^ = 0.22; Index = −0.002, *SE* = 0.02, 95% CI [−0.04, 0.04]. Thus, this hypothesis was not supported.

We also tested whether processing fluency condition would indirectly influence bias using the same analysis as above (Table 4). Across this analysis, the indirect effects on bias were not distinguishable from zero – message conditions, *B* = −0.01, *SE* = 0.01, 95% CI [−0.03, 0.01]; listing conditions, *B* = −0.03, *SE* = 0.03, 95% CI [−0.09, 0.02], *R^2^* = 0.12. This suggests that processing fluency condition did not indirectly impact bias.

**Table 4:** Serial mediation results predicting bias.

Predictors	Path 1 *B* (*SE*)	Path 2 *B* (*SE*)	Path 3 *B* (*SE*)
Intercept	2.88 (0.43)***	1.23 (0.37)***	0.11 (0.21)
Processing fluency condition	0.56 (0.12)***	−0.10 (0.11)	0.13 (0.06)*
Task condition	–0.25 (0.13)*	0.24 (0.11)*	0.15 (0.06)*
PF × Task conditions	1.24 (0.18)***	−0.50 (0.15)**	−0.22 (0.09)*
Processing fluency		0.18 (0.03)***	−0.02 (0.02)
Internal efficacy			−0.06 (0.02)***
Faith in intuition for facts	0.06 (0.05)	0.26 (0.05)***	0.04 (0.03)
Need for evidence	0.26 (0.07)***	0.38 (0.06)***	−0.09 (0.03)**
Truth is political	–0.09 (0.05)	0.08 (0.04)	0.01 (0.02)
Conspiracy mentality	–0.02 (0.04)	−0.01 (0.03)	0.03 (0.02)
Political ideology	–0.01 (0.03)	0.07 (0.02)**	0.01 (0.01)
*F*	33.44***	14.86***	5.75***
*R^2^*	0.21	0.12	0.06

Path 1 indicates the unstandardized relationship between processing fluency condition (0: difficult, 1: easy) and self-reported processing fluency. Path 2 indicates the unstandardized relationship between self-reported processing fluency and internal efficacy. Path 3 indicates the unstandardized relationship between internal efficacy and bias (ln[β]). *p < 0.05, **p < 0.01, ***p < 0.001.

Hypothesis five predicted that internal efficacy would be a significant predictor of accuracy. Overall, this component of the model was significant, *F*(10, 989) = 28.41, *p* < 0.001, *R^2^* = 0.22, as internal efficacy was a positive predictor of *d’* (*B* = 0.05, *SE* = 0.02, *t* = 2.87, *p* < 0.01) even when covarying participants’ faith in intuition, need for evidence, truth is political, conspiracy mentality, and political ideology (Table 3, Path 3). This *d’* coefficient can be interpreted as participants improved ability to accurately discriminate hits (facts) from foils (misinformation). This result suggests that gains in internal efficacy improved misinformation detection. We also tested this model with bias as the outcome. In this model, *F*(10, 989) = 5.75, *p* < 0.001, *R^2^* = 0.06, efficacy was a negative predictor of bias (*B* = −0.06, *SE* = 0.02, *p* < 0.001). This suggests that as efficacy increased, people registered more of a truth bias. Taken together, these outcomes were consistent with H5 which predicted that efficacy should impact accuracy.

Hypothesis six predicted that there would be a positive indirect effect of the easy condition on accuracy through the mediators of processing fluency and internal efficacy. A positive, non-zero indirect effect was found at both levels of the moderator (task condition) – message conditions, *B* = 0.01, *SE* = 0.002, 95% CI [0.002, 0.01]; listing conditions, *B* = 0.02, *SE* = 0.01, 95% CI [0.01, 0.03], *R*^2^ = 0.22; Index = 0.01, *SE* = 0.005, 95% CI [0.004, 0.02] (see Table 3 and Figure 2 for full results). These results suggest that increased levels of processing fluency, evoked through the processing fluency manipulations, and gains in internal efficacy, increased accuracy. Thus, H6 was supported.

**Figure 4:**
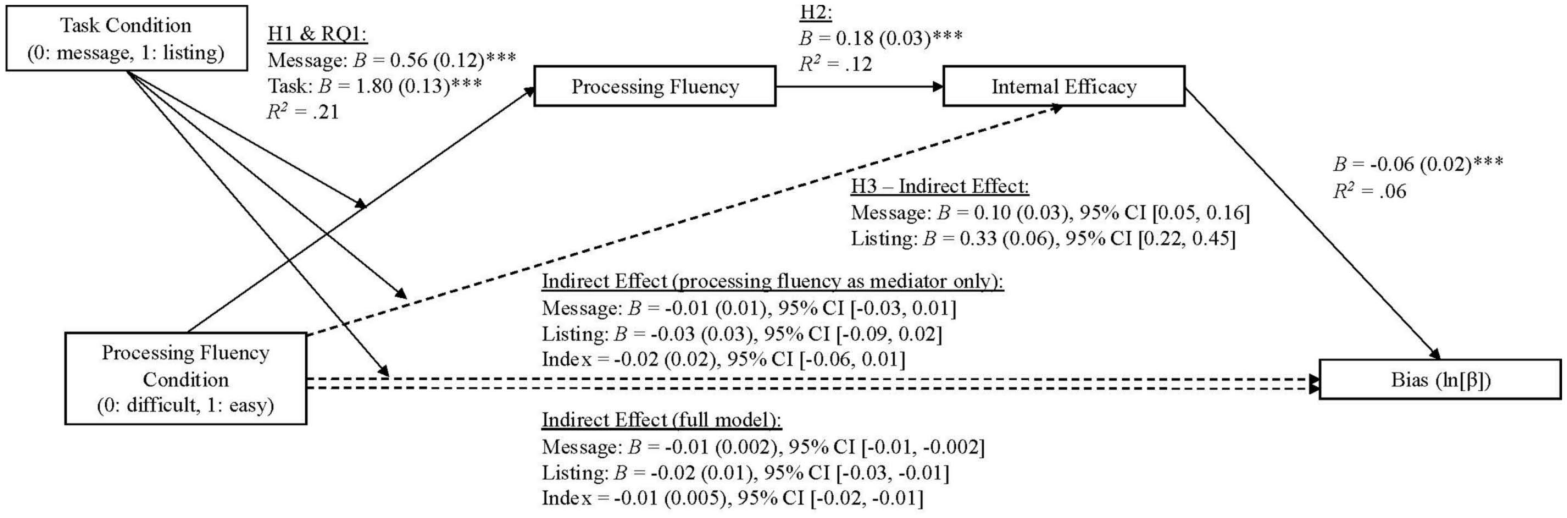
Serial mediation model predicting bias. Standard errors are shown in parentheses. Index refers to the index of moderated mediation. Participants’ epistemic beliefs, conspiracy mentality, and political ideology were included as covariates in the model. For all analyses, mediation is supported if the 95% confidence interval does not include zero. ***p < 0.001.

We also examined whether there would be an indirect effect of processing fluency condition on bias through the serial mediators of processing fluency and internal efficacy, using the same analysis as above (full results available in Table 4 and Figure 4). We found negative, non-zero indirect effect of the processing fluency condition at both levels of the moderator (task type)-message conditions, *B* = −0.01, *SE* = 0.002, 95% CI [−0.01, −0.002]; listing conditions, *B* = −0.02, *SE* = 0.01, 95% CI [−0.03, −0.01]; *R^2^* = 0.06; Index, *B* = −0.01, *SE* = 0.005, 95% CI [−0.02, −0.01]. Together, these results suggest that increased levels of processing fluency, evoked through the processing fluency task manipulations, and gains in internal efficacy, increased truth bias.

## Discussion

The purpose of this experiment was to investigate the effects of a state of processing fluency on one’s propensity to detect misinformation. We proposed that, in online contexts, readers are likely to scan a variety of information, from a variety of contexts, on a variety of topics. Our thinking was that rather than isolate any singular message feature (e.g., repetition) or any particular individual difference characteristic (e.g., conservativism), we would instead seek to understand how a state of fluency might impact information processing in general. As we argued throughout, a state of processing fluency has been associated with both tacit message acceptance (Petty et al., 2007; Schwarz, 2011), as well as increased message scrutiny (Petty et al., 2002; Tormala et al., 2002). Thus, the question we posed here is whether processing fluency improves or degrades one’s ability to detect misinformation. In this section, we explain our findings, theoretical contributions, practical implications, and the limitations of this effort.

### Summary of findings

We set out to investigate whether a state of processing fluency, as opposed to processing fluency evoked through a particular message feature, would increase the likelihood that people would believe misinformation. To address this question, we used two different manipulations of processing fluency. Thus, our first hypothesis (and RQ) intended to replicate prior work by testing whether a message featuring difficult language, or a task that required participants to list more (versus less) exemplars, would impair reports of processing fluency relative to the easier versions of the stimuli/task. Consistent with our intent, we found that participants in the easy conditions of both the message and listing tasks reported higher levels of processing fluency than participants in the difficult message and listing conditions. Thus, a state of processing fluency, before engaging in the misinformation detection task, was successfully induced. That said, the thought-listing task produced a stronger manipulation of processing fluency than the message task (RQ1). Perhaps this suggests that more active forms of this manipulation (asking people to generate a list) versus more passive forms (reading a text) can make the metacognitive feelings that accompany these experiences more potent. This active approach is something to be mindful of when considering how our findings, discussed below, can be applied to combat misinformation.

We focused on inducing a state of fluency, before exposure to the misinformation task because processing fluency has been shown to be associated with perceptions about the state of one’s knowledge (Petty et al., 2007). Specifically, an easier experience enhances perceptions of knowledge, abilities, and confidence whereas a difficult experience degrades these perceptions (see Petty et al., 2002; Petty et al., 2007; Shulman and Sweitzer, 2018). Thus, processing fluency should positively associate with internal efficacy. Consistent with the extant literature, in this experiment processing fluency was significantly positively associated with internal efficacy (H2) and our processing fluency manipulation was significantly and positively indirectly associated with internal efficacy through processing fluency (H3), as well. These findings are consistent with the self-validation hypothesis (Petty et al., 2002; Tormala et al., 2002), which contends that an easy experience can instill confidence in one’s thoughts. In the persuasion literature, this confidence has been shown to impact persuasive outcomes and thus should be taken into account when trying to understand how various cognitions and metacognitions come to impact beliefs, attitudes, and behaviors.

Given these ideas, we next set out to ascertain how processing fluency, and its correlates, might be associated with accuracy in evaluating fact-based information. As we see it, the dual-process and metacognition literatures suggest two possible relationships between processing fluency and accuracy. The first perspective would suggest that increases in processing fluency should be associated with an increased *yes*, or truth bias, as people should be more likely to accept new information encountered under a state of fluency (Reber and Schwarz, 1999; Markowitz and Hancock, 2016; McGlone and Tofighbakhsh, 2000; Brennan and Williams, 1995). This possibility is consistent with articles suggesting that processing fluency produces a more heuristic style of processing (e.g., Shulman et al., 2022). Thus, this perspective would suggest that a state of fluency should impair the detection of misinformation. The results of H4, however, were not significant, suggesting that a state of fluency did not seem to impact accuracy in detecting misinformation.

The second perspective we tested was whether processing fluency could cue more systematic processing. One of the naïve theories that underlie FIT (Schwarz, 2011), and the self-validation hypothesis (Petty et al., 2002), is the idea that processing fluency is diagnostic about the state of one’s knowledge. Specifically, easier experiences increase feelings of knowledge and confidence in one’s ability (captured here through the concept of efficacy), whereas more difficult experiences lead to deficits in these categories. This is the idea that underlies the self-validation hypothesis (see Petty et al., 2002) which argues that enhancements to self-confidence will increase the systematic processing of information. We tested this hypothesis by examining whether perceptual gains in efficacy correspond with actual gains in one’s ability to discern misinformation from factual information. We found that gains in internal efficacy were positively associated with accuracy and negatively associated with bias (increased truth bias) (H5). Further, we found support for our larger serial mediation model (H6) that linked assignment to an easy processing fluency condition to increased misinformation detection through self-reported processing fluency and internal efficacy. This finding indicates that if an individual’s internal efficacy is increased via a state of fluency, they may be more discerning and accurate when assessing information. This has important implications for combatting misinformation. Specifically, these findings imply that that external manipulations of fluency, like providing users with an easy processing experience or boosting self-confidence, may prime a more systematic, and discerning, processing style. We elaborate on these ideas below.

### Theoretical contributions

The theoretical goal of this work was to understand how cognitive states, beyond individual differences, message features, or motivated reasoning, may contribute to the acceptance of misinformation. Given that this is one of the first investigations that links fluency and efficacy to misinformation detection, this study can be considered a “proof of concept” or an initial test of whether fluency and efficacy can impact how carefully people scrutinize information. Practically, our results suggest that increasing people’s efficacy, or belief that they are able to discern high quality from low quality information, can impact people’s actual ability to do so. Theoretically, our research contributes by proffering that fluency and efficacy can produce a motivational state that improves misinformation detection. This finding is noteworthy given that the broader literature on metacognition has debated about whether a state of fluency produces more heuristic versus systematic processing (see Petty et al., 2002; Petty et al., 2007; Schwarz and Lee, 2017; Shulman and Bullock, 2020; Shulman et al., 2022). Our findings suggest that, when coupled with internal efficacy, increases in fluency can help increase the degree to which people scrutinize information. Thus, support is offered for the role of fluency as a potential motivator of systematic processing.

Importantly, when integrating these claims within the broader literature on misinformation, our findings imply that interventions that boost people’s efficacy, or confidence, in their ability to scrutinize information may reduce misinformation acceptance. This intervention strategy presents a notable departure from the more common interventions, such as fact-checking. Given that fact-checking misinformation has received modest support at best (Walter et al., 2020), perhaps taking a more efficacy-based approach, and using fluency to enhance these perceptions, can be a fruitful way of combatting misinformation moving forward. In light of these findings, we suggest more work in this area is both needed and warranted.

### Limitations and conclusion

Although our results offer promising insights into potential ways to combat misinformation acceptance, there are limitations and open questions that should be addressed with future work. First, we acknowledge that there is reasonable question as to whether our processing fluency manipulation positively impacted internal efficacy or if our misinformation detection task influenced efficacy reports. This remains an empirical question because we measured efficacy after participants saw and responded to the misinformation statements. Thus, it is possible that an easier performance on the detection task increased feelings of efficacy, and not the other way around. Before launching this experiment, we intentionally chose to place efficacy items after the misinformation task because the naïve theory literature tells us that naïve theories only operate when attention is not drawn to their functioning (Schwarz and Clore, 2013). We were concerned that placing measures about internal efficacy directly before the misinformation task could prime participants into trying harder on the task. This would then be an efficacy induction and not a processing fluency induction, which was not the purpose of this study (though rife for future work). Moreover, we contend that including efficacy items after the misinformation task was justifiable given that much work has documented a positive relationship between processing fluency and self-efficacy in a variety of domains (Kim and Jang, 2018; Okuhara et al., 2020; Petty et al., 2007; Schwarz, 2015; Shulman and Bullock, 2019). Despite this theoretical precedent, however, our findings should be interpreted with skepticism until a follow-up investigation can more fully resolve the causal relationship between fluency, efficacy, and information processing. Moving forward, this limitation could be attended to by randomizing the order of appearance between the efficacy scale and signal detection task or through a closer examination of the causal relationships between processing fluency inductions, processing fluency reports, and internal efficacy in a separate experiment.

In addition to this order issue, we also acknowledge a potential confound in our results related to the two task conditions. Specifically, while participants in the message condition read about technology regulations, participants in the listing conditions recalled technology leaders. Although these topics are both about the technology industry, it is possible that slight differences in topic (leaders vs. regulation) could have contributed to the differences observed in processing fluency reports. Future work should continue to investigate the differences in fluency inductions, with special attention paid towards achieving consistency across manipulations, and continuing to parse out the causal order of these variables. Finally, we note that our signal detection measures of accuracy (*d’*) and bias (β) are significantly correlated (*r* = −0.606, *p* < 0.001), suggesting that one or both of the statistical assumptions (normality and equal variances) was violated (Stanislaw and Todorov, 1999). Given this possibility, it becomes important to replicate these findings with future work.

In sum, this experiment’s purpose was to advance theory and explore whether processing fluency can influence the ability to accurately discriminate between true and false information. Our results suggest that increased processing fluency is associated with an increase in internal efficacy, which then resulted in more detection accuracy. Our unique approach to manipulating processing fluency external to a message and the use of short, varied fact-based statements was meant to approximate a person’s state of mind as they process information in online environments (Pearson, 2021). Our work suggests that if individuals are primed with a state of fluency that enhances their domain specific self-efficacy, they are more likely to engage in the systematic processing of information, at least when explicitly asked about the information’s validity. These results provide insight into the cognitions that support the acceptance of misinformation (disfluency and reductions in confidence) and begins to consider novel cognitive-based solutions for how to combat the spread of misinformation.

## Data Availability

The raw data supporting the conclusions of this article will be made available by the authors, without undue reservation.

## Footnotes

^1^The Flesch–Kincaid grade level assesses readability based on a U.S. grade school education level (i.e., a score of 8.0 means an eighth grader can comprehend the text). This score is calculated using the average number of words per sentence and the average number of syllables per word [*NL 10605.105–What is the Flesch-Kincaid Readability Test?* (Social Security Administration, 2015)].

^2^We investigated whether these covariates could be classified as individual differences and thus unimpacted by the experimental manipulations. To test this, we ran two-way ANOVAs to examine whether the variables were influenced by the processing fluency manipulation, the task, or the interaction effect. There were no significant effects between the tasks on these variables (three epistemic beliefs sub-scales, conspiratorial mentality, and political ideology). Thus, these variables were included in all tests as covariates so that their influence on misinformation detection could be taken into account, and thus accounted for, alongside our experimental manipulations.

## References

[ref1] Alter A. L. Oppenheimer D. M. (2009). Uniting the tribes of fluency to form a metacognitive nation. Personal. Soc. Psychol. Rev. 13, 219–235. doi: 10.1177/1088868309341564, PMID: 19638628

[ref2] Alter A. L. Oppenheimer D. M. Epley N. Eyre R. N. (2007). Overcoming intuition: metacognitive difficulty activates analytic reasoning. J. Exp. Psychol. Gen. 136, 569–576. doi: 10.1037/0096-3445.136.4.569 17999571

[ref3] Aslett K. Sanderson Z. Godel W. Persily N. Nagler J. Tucker J. A. (2024). Online searches to evaluate misinformation can increase its perceived veracity. Nature 625, 548–556. doi: 10.1038/s41586-023-06883-y 38123685 PMC10794132

[ref5] Bacon F. T. (1979). Credibility of repeated statements: memory for trivia. J. Exp. Psychol. Hum. Learn. Mem. 5, 241–252.

[ref6] Barchetti A. Neybert E. Mantel S. P. Kardes F. R. (2022). The half-truth effect and its implications for sustainability. Sustain. For. 14:6943. doi: 10.3390/su14116943

[ref7] Batailler C. Brannon S. M. Teas P. E. Gawronski B. (2022). A signal detection approach to understanding the identification of fake news. Perspect. Psychol. Sci. 17, 78–98. doi: 10.1177/1745691620986135 34264150

[ref8] Baughan A. Zhang M. R. Rao R. Lukoff K. Schaadhardt A. Butler L. D.. (2022). “I don’t even remember what I read”: how design influences dissociation on social media. CHI Conf. Hum. Factors Comput. Syst., 1–13. doi: 10.1145/3491102.3501899

[ref9] Begg I. M. Anas A. Farinacci S. (1992). Dissociation of processes in belief: source recollection, statement familiarity, and the illusion of truth. J. Exp. Psychol. Gen. 121, 446–458.

[ref10] Bishop G. F. Oldendick R. W. Tuchfarber A. (1984). What must my interest in politics be if I just told you “I don’t know”? Public Opin. Q. 48, 510–519.

[ref11] Boehm L. E. (1994). The validity effect: a search for mediating variables. Personal. Soc. Psychol. Bull. 20, 285–293. doi: 10.1177/0146167294203006

[ref12] Brennan S. E. Williams M. (1995). The feeling of another’s knowing: prosody and filled pauses as cues to listeners about the metacognitive states of speakers. J. Mem. Lang. 34, 383–398.

[ref13] Bruder M. Haffke P. Neave N. Nouripanah N. Imhoff R. (2013). Measuring individual differences in generic beliefs in conspiracy theories across cultures: conspiracy mentality questionnaire. Front. Psychol. 4:225. doi: 10.3389/fpsyg.2013.00225, PMID: 23641227 PMC3639408

[ref14] Bullock O. M. Colón Amill D. Shulman H. C. Dixon G. N. (2019). Jargon as a barrier to effective science communication: evidence from metacognition. Public Underst. Sci. 28, 845–853. doi: 10.1177/0963662519865687 31354058

[ref15] Bullock O. M. Shulman H. C. Huskey R. (2021). Narratives are persuasive because they are easier to understand: examining processing fluency as a mechanism of narrative persuasion. Front. Commun. 6:719615. doi: 10.3389/fcomm.2021.719615

[ref9001] Domgaard S. Park M. (2021). Combating misinformation: the effects of infographics in verifying false vaccine news. Health Educ J 80, 974–986. doi: 10.1177/00178969211038750

[ref16] Eagly A. Chaiken S. (1993). The psychology of attitudes: Harcourt Brace Jovanovich College Publishers. (Fort Worth, TX: Eagly & Chaiken).

[ref17] Fallis D. (2015). What is disinformation? Libr. Trends 63, 401–426. doi: 10.1353/lib.2015.0014

[ref18] Flynn D. J. Nyhan B. Reifler J. (2017). The nature and origins of misperceptions: understanding false and unsupported beliefs about politics. Polit. Psychol. 38, 127–150. doi: 10.1111/pops.12394

[ref19] Garrett R. K. Bond R. M. (2021). Conservatives’ susceptibility to political misperceptions. Science. Advances 7:eabf1234. doi: 10.1126/sciadv.abf1234, PMID: 34078599 PMC8172130

[ref20] Garrett R. K. Weeks B. E. (2017). Epistemic beliefs’ role in promoting misperceptions and conspiracist ideation. PLoS One 12:e0184733. doi: 10.1371/journal.pone.0184733 28922387 PMC5603156

[ref21] Hasher L. Goldstein D. Toppino T. (1977). Frequency and the conference of referential validity. J. Verbal Learn. Verbal Behav. 16, 107–112. doi: 10.1016/S0022-5371(77)80012-1

[ref22] Hayes A. F. (2009). Beyond baron and Kenny: statistical mediation analysis in the new millennium. Commun. Monogr. 76, 408–420. doi: 10.1080/03637750903310360

[ref23] Hayes A. F. (2013). Introduction to mediation, moderation, and conditional process analysis: a regression-based approach. New York, NY: Guilford Press.

[ref24] Heider F. (1958). The psychology of interpersonal relations Ed. Lawrence. Hillsdale, NJ. (New York, NY: Erlbaum Associates, Inc., Wiley).

[ref25] Kahneman D. (2011). Thinking, fast and slow. 1st Edn. New York, NY: Farrar, Straus, and Giroux.

[ref26] Kim H. Jang J. (2018). The easier the better: how processing fluency influences self-efficacy and behavioral intention in pro-social campaign advertising. Sustain. For. 10:4777. doi: 10.3390/su10124777

[ref27] Klar R. (2023). Democrats urge Biden to follow Europe’s lead on strict tech regulations: The Hill. Available at: https://thehill.com/business/4358437-democrats-urge-biden-to-follow-europes-lead-on-strict-tech-regulations/ (Accessed October 4, 2024).

[ref28] Levine T. R. (2019). Duped: Truth-default theory and the social science of lying and deception. Tuscaloosa, AL: University of Alabama Press.

[ref29] Loomba S. De Figueiredo A. Piatek S. J. De Graaf K. Larson H. J. (2021). Measuring the impact of COVID-19 vaccine misinformation on vaccination intent in the UK and USA. Nat. Hum. Behav. 5, 337–348. doi: 10.1038/s41562-021-01056-1 33547453

[ref30] Markowitz D. M. Hancock J. T. (2016). Linguistic obfuscation in fraudulent science. J. Lang. Soc. Psychol. 35, 435–445. doi: 10.1177/0261927X15614605

[ref31] Markus H. (1977). Self-schemata and processing information about the self. J. Pers. Soc. Psychol. 35, 63–78.

[ref32] McCarthy B. (2021). How election misinformation fueled the Jan. 6 insurrection. PolitiFact. Available at: https://www.politifact.com/article/2021/jun/30/misinformation-and-jan-6-insurrection-when-patriot/ (Accessed April 12, 2024).

[ref33] McGlone M. S. Tofighbakhsh J. (2000). Birds of a feather flock conjointly (?): rhyme as reason in aphorisms. Psychol. Sci. 11, 424–428. doi: 10.1111/1467-9280.00282 11228916

[ref34] Miele D. B. Molden D. C. (2010). Naive theories of intelligence and the role of processing fluency in perceived comprehension. J. Exp. Psychol. Gen. 139, 535–557. doi: 10.1037/a0019745 20677898

[ref35] Newman E. J. (2013). Nonprobative photos inflate the truthiness and falsiness of claims. (Wellington, NZ: Victoria University of Wellington).

[ref36] Newman E. J. Garry M. Bernstein D. M. Kantner J. Lindsay D. S. (2012). Nonprobative photographs (or words) inflate truthiness. Psychon. Bull. Rev. 19, 969–974. doi: 10.3758/s13423-012-0292-0 22869334

[ref37] Newman E. J. Zhang L. (2021). “Truthiness: how non-probative photos shape belief” in The psychology of fake news. eds. Greifeneder R. Jaffe M. Newman E. Schwarz N.. 1st ed (New York, NY: Routledge), 90–114.

[ref38] Niemi R. G. Craig S. C. Mattei F. (1991). Measuring internal political efficacy in the 1988 National Election Study. Am. Polit. Sci. Rev. 85, 1407–1413. doi: 10.2307/1963953

[ref39] O’Keefe D. J. (2003). Message properties, mediating states, and manipulation checks: claims, evidence, and data analysis in experimental persuasive message effects research. Commun. Theory 13, 251–274. doi: 10.1111/j.1468-2885.2003.tb00292.x

[ref40] Okuhara T. Ishikawa H. Ueno H. Okada H. Kato M. Kiuchi T. (2020). Influence of high versus low readability level of written health information on self-efficacy: a randomized controlled study of the processing fluency effect. Health Psychol Open 7, 1–9. doi: 10.1177/2055102920905627 PMC7016314 32110424

[ref41] Oppenheimer D. M. (2006). Consequences of erudite vernacular utilized irrespective of necessity: problems with using long words needlessly. Appl. Cogn. Psychol. 20, 139–156. doi: 10.1002/acp.1178

[ref42] Oppenheimer D. M. (2008). The secret life of fluency. Trends Cogn. Sci. 12, 237–241. doi: 10.1016/j.tics.2008.02.014, PMID: 18468944

[ref43] Pearson G. (2021). Sources on social media: information context collapse and volume of content as predictors of source blindness. New Media Soc. 23, 1181–1199. doi: 10.1177/1461444820910505

[ref44] Pearson G. D. H. Cappella J. N. (2024). Scrolling past public health campaigns: information context collapse on social media and its effects on tobacco information recall. Int. J. Commun. 18, 1112–1134.

[ref45] Petty R. E. Briñol P. Tormala Z. L. (2002). Thought confidence as a determinant of persuasion: the self-validation hypothesis. J. Pers. Soc. Psychol. 82, 722–741. doi: 10.1037/0022-3514.82.5.722 12003473

[ref46] Petty R. Briñol P. Tormala Z. L. Wegener D. T. (2007). “The role of metacognition in social judgment” in Social psychology: handbook of basic principles. eds. Kruglanski A. W. Higgings E. T.. 2nd ed (New York, NY: Guilford Press), 254–284.

[ref47] Petty R. E. Cacioppo J. T. (1986). The elaboration likelihood model of persuasion. Adv. Exp. Soc. Psychol. 19, 123–201. doi: 10.1016/S0065-2601(08)60214-2

[ref48] Pew Research Center. (2023). News platform fact sheet [pew research center]. Pew Research Center’s Journalism Project. Available at: https://www.pewresearch.org/journalism/fact-sheet/news-platform-fact-sheet/ (Accessed April 4, 2024).

[ref49] Priedols M. Dimdins G. (2023). Evaluation of misinformation among pro-Ukrainian Latvians – the role of prior attitude, analytical thinking, and emotions. Front. Psychol. 14:1165039. doi: 10.3389/fpsyg.2023.1165039 37780159 PMC10538560

[ref50] Reber R. Schwarz N. (1999). Effects of perceptual fluency on judgments of truth. Conscious. Cogn. 8, 338–342. doi: 10.1006/ccog.1999.0386 10487787

[ref51] Reber R. Zupanek N. (2002). “Effects of processing fluency on estimates of probability and frequency” in Etc. frequency processing and cognition. eds. Sedlmeier P. Betsch T. (Oxford: Oxford University Press).

[ref52] Riggs E. E. Shulman H. C. Lopez R. (2022). Using infographics to reduce the negative effects of jargon on intentions to vaccinate against COVID-19. Public Underst. Sci. 31, 751–765. doi: 10.1177/09636625221077385 35266427

[ref53] Sanchez G. R. Middlemass K. (2022). Misinformation is eroding the public’s confidence in democracy. Brookings. Available at: https://www.brookings.edu/articles/misinformation-is-eroding-the-publics-confidence-in-democracy/ (Accessed April 2, 2024).

[ref9002] Schwarz N. (2004). Metacognitive experiences in consumer judgment and decision making. J. Consum. Psychol 14, 332–348. doi: 10.1207/s15327663jcp1404_2

[ref54] Schwarz N. (2006). Feelings, fit, and funny effects: a situated cognition perspective. J. Mark. Res. 43, 20–23. doi: 10.1509/jmkr.43.1.20

[ref55] Schwarz N. (2011). Feelings-as-information theory. In Lange P. Van Kruglanski A. Higgins E. (Eds.), Handbook of theories of social psychology (Vol. 1, pp. 289–308). Eds. P. Van Lange. (Los Angeles, CA: SAGE Publications Ltd).

[ref56] Schwarz N. (2015). “Metacognition” in APA handbook of personality and social psychology. eds. Mikulincer M. Shaver P. R. Borgida E. Bargh J. A., vol. 1 (Washington, D.C.: American Psychological Association), 203–229.

[ref57] Schwarz N. Bless H. Strack F. Klumpp G. Rittenauer-Schatka H. Simons A. (1991). Ease of retrieval as information: another look at the availability heuristic. J. Pers. Soc. Psychol. 61, 195–202.

[ref58] Schwarz N. Clore G. L. (2013). “Feelings and phenomenal experiences” in Social psychology: Handbook of basic principles. eds. Kruglanski A. W. Higgins E. T., vol. 2 (New York, NY: Guilford), 385–407.

[ref9003] Schwarz N. Schuman H. (1997). Political knowledge, attribution, and inferred interest in politics: The operation of buffer items. I*nt J Public Opin Res* 9, 191–195. doi: 10.1093/ijpor/9.2.191

[ref59] Schwarz N. Lee S. W. S. (2017). “Embodied cognition and the construction of attitudes” in Handbook of attitudes. eds. Albarracín D. Johnson B. T.. 2nd ed (New York, NY: Routledge).

[ref60] Shulman H. C. Bullock O. M. (2019). Using metacognitive cues to amplify message content: a new direction in strategic communication. Ann. Int. Commun. Assoc. 43, 24–39. doi: 10.1080/23808985.2019.1570472

[ref61] Shulman H. C. Bullock O. M. (2020). Don’t dumb it down: the effects of jargon in COVID-19 crisis communication. PLoS One 15:e0239524. doi: 10.1371/journal.pone.0239524 33027268 PMC7540871

[ref62] Shulman H. C. Dixon G. N. Bullock O. M. Colón Amill D. (2020). The effects of jargon on processing fluency, self-perceptions, and scientific engagement. J. Lang. Soc. Psychol. 39, 579–597. doi: 10.1177/0261927X20902177

[ref63] Shulman H. C. Sweitzer M. D. (2018). Varying metacognition through public opinion questions: how language can affect political engagement. J. Lang. Soc. Psychol. 37, 224–237. doi: 10.1177/0261927X17707557

[ref64] Shulman H. C. Sweitzer M. D. Bullock O. M. Coronel J. C. Bond R. M. Poulsen S. (2022). Predicting vote choice and election outcomes from ballot wording: the role of processing fluency in low information direct democracy elections. Polit. Commun. 39, 652–673. doi: 10.1080/10584609.2022.2092920

[ref65] Slater M. D. Gleason L. S. (2012). Contributing to theory and knowledge in quantitative communication science. Commun. Methods Meas. 6, 215–236. doi: 10.1080/19312458.2012.732626

[ref66] So J. Song H. (2023). Two faces of message repetition: audience favorability as a determinant of the explanatory capacities of processing fluency and message fatigue. J. Commun. 73, 574–586. doi: 10.1093/joc/jqad025

[ref4] Social Security Administration (2015). NL 10605.105—What is the Flesch-Kincaid readability test? Available at: https://secure.ssa.gov/poms.nsf/lnx/0910605105 (Accessed July 3, 2024).

[ref67] Song H. Schwarz N. (2008a). Fluency and the detection of misleading questions: low processing fluency attenuates the moses illusion. Soc. Cogn. 26, 791–799. doi: 10.1521/soco.2008.26.6.791

[ref68] Song H. Schwarz N. (2008b). If it’s hard to read, it’s hard to do: processing fluency affects effort prediction and motivation. Psychol. Sci. 19, 986–988. doi: 10.1111/j.1467-9280.2008.02189.x 19000208

[ref69] Song H. Schwarz N. (2009). If it’s difficult to pronounce, it must be risky: fluency, familiarity, and risk perception. Psychol. Sci. 20, 135–138. doi: 10.1111/j.1467-9280.2009.02267.x 19170941

[ref70] Stanislaw H. Todorov N. (1999). Calculation of signal detection theory measures. Behav. Res. Methods Instrum. Comput. 31, 137–149. doi: 10.3758/BF03207704 10495845

[ref71] Susmann M. W. Wegener D. T. (2023). How attitudes impact the continued influence effect of misinformation: the mediating role of discomfort. Personal. Soc. Psychol. Bull. 49, 744–757. doi: 10.1177/01461672221077519 35227114

[ref72] Tolochko P. Song H. Boomgaarden H. (2019). “That looks hard!”: effects of objective and perceived textual complexity on factual and structural political knowledge. Polit. Commun. 36, 609–628. doi: 10.1080/10584609.2019.1631919

[ref73] Tormala Z. L. Petty R. E. Briñol P. (2002). Ease of retrieval effects in persuasion: a self-validation analysis. Personal. Soc. Psychol. Bull. 28, 1700–1712. doi: 10.1177/014616702237651

[ref74] Tversky A. Kahneman D. (1973). Availability: a heuristic for judging frequency and probability. Cogn. Psychol. 5, 207–232.

[ref75] Vegetti F. Mancosu M. (2020). The impact of political sophistication and motivated reasoning on misinformation. Polit. Commun. 37, 678–695. doi: 10.1080/10584609.2020.1744778

[ref76] Walter N. Cohen J. Holbert R. L. Morag Y. (2020). Fact-checking: a meta-analysis of what works and for whom. Polit. Commun. 37, 350–375. doi: 10.1080/10584609.2019.1668894

[ref77] Wegener D. T. Petty R. E. (1998). The naive scientist revisited: naive theories and social judgment. Soc. Cogn. 16, 1–7. doi: 10.1521/soco.1998.16.1.1

