# Supplementary material for: Using a signal detection approach to understand the impacts of processing fluency and efficacy on accuracy in misinformation detection
Source: Front Psychol. 2024 Sep 16;15:1417910. doi: 10.3389/fpsyg.2024.1417910 (PMC11443571; doi:10.3389/fpsyg.2024.1417910)
Supplement: Supplementary file 1 [file Data_Sheet_1.PDF]

Supplementary Materials for

Using a Signal Detection Approach to Understand the Impacts of Processing Fluency and Efficacy on Accuracy in Misinformation Detection

TABLE OF CONTENTS

Experimental Stimuli ***3***

Message Condition: Easy **3**

Message Condition: Difficult **3**

Listing Task (i.e., Retrieval Task) Conditions **4**

Measures ***5***

Processing Fluency **5**

Internal Efficacy **5**

Misinformation Statements, Accuracy, Fact-Check Cite (where relevant) **5**

Epistemic Beliefs Scales **6**

Conspiracy Mentality Scale **7**

Political Ideology **7**

Supplementary Tables and Figure ***8***

Supplementary Table S1. ***Correlation Matrix*** **9**

EXPERIMENTAL STIMULI

MESSAGE CONDITION: EASY

Democrats asked President Biden to take up trade policy that supports the European Union's new policies. These policies would target the market power of tech giants. It would also reject claims that the rules are bad for trade, according to a letter sent Wednesday.

The letter supports the EU's Digital Markets ACT (DMA), which adds guidelines for large tech companies. The DMA went into law in May.

"We ask you to stop saying that the European Union's Digital Markets Act (DMA) is bad for trade. The DMA will protect customers. It will create competition in tech. The United States' trade policy must support the European Union's efforts to control Big Tech. We must support similar American policies, not stop them," the Democrats wrote.

The lawmakers who sent the letter blamed the tech giants, and their trade groups along with "their allies in government." The lawmakers said the tech companies were wrong in saying that the DMA will be bad for trade.

"The DMA is fair because it does not target companies, platforms, services, investors, or digital products based on where they are from. Instead the DMA will help regulate selfish behavior by the largest companies in the digital market," they wrote.

"And if the DMA impacts mostly large tech companies in the United States, it is because they have participated in control tactics to gain industry power, not to help America," they added.

The Democrats praised Biden's work pushing to control the power of tech companies. For example, the Department of Justice's antitrust lawsuits against Google. They urged the President to "remain strong in the face of Big Tech's biased statements."

MESSAGE CONDITION: DIFFICULT

Democrats urged President Biden to pursue trade policy that supports the European Union's new regulations targeting the market power of tech giants, and to falsify claims that the rules create barriers to trade, according to a letter sent Wednesday.

The letter articulates support for the EU's Digital Markets ACT (DMA), which adds supplementary regulations for large tech companies and became legislation in May.

"We urge you to continue to reject claims that the European Union's Digital Markets Act (DMA) constitutes an illegal barrier to trade. The DMA will protect consumers and spur tech competition. The United States' trade policy must reify the European Union's efforts to rein in Big Tech and facilitate similar American policies, rather than impair them," the Democrats wrote.

The lawmakers who sent the letter accused the tech giants, their associated trade groups and “their allies in government,” of “erroneously” claiming that the DMA is an illegal trade barrier.

“The DMA is not discriminatory because it does not target companies, platforms, services, investors or digital products by their national origin. Rather, it establishes a framework for regulating monopolistic behavior by the largest firms in the digital market,” they wrote.

“And if the effect of the DMA lands predominantly on massive tech corporations based in the United States, it is because they have engaged in anticompetitive tactics to achieve industry dominance, not because they are American,” they added.

The Democrats praised Biden’s work to rein in the power of tech companies, for example, through the Department of Justice’s antitrust lawsuits against Google. They urged the president to “remain steadfast in the face of Big Tech’s misleading trade discrimination claims.”

Listing Task (i.e., Retrieval Task) Conditions

Prompt: Please do not look up the answers to this task.

1. Easy task: Please list 2 leaders of technology companies.
2. Hard task: Please list 8 leaders of technology companies.

Measures

Processing Fluency

Answered on a 7-point scale from strongly disagree to strongly agree.

1. The ideas presented in this article felt new to me. (RC)
2. Overall, I found this article difficult to read. (RC)
3. It was easy for me to think about the ideas in the article.
4. The article felt easy to read.
5. The article felt hard to read. (RC)
6. It felt like it took a long time to read the article. (RC)

Internal Efficacy

1. I consider myself to be well qualified to participate in current events.
2. I feel like I have a pretty good understanding of the important current issues facing our country.
3. I feel that I could do as good a job in public office as most other people.
4. I think that I am better informed about current events than most people.

Misinformation Statements, Accuracy, Fact-Check Cite (where relevant)

1. Republican presidential candidate Nikki Haley supports a 23% national sales tax. (False - https://www.factcheck.org/2024/02/trump-haley-trade-false-and-misleading-attacks/)
2. None of the classified documents found in President Biden’s possession were highly classified. (False - https://www.politifact.com/factchecks/2024/feb/09/joe-biden/president-joe-biden-said-he-didnt-have-highly-clas/)
3. Donald Trump deported less people than Barack Obama did during his presidency. (True - https://www.politifact.com/factchecks/2024/jan/04/ron-desantis/ron-desantis-is-right-barack-obama-deported-more-p/)
4. Postpartum Medicaid coverage expanded from three states to 43 states because of the Biden administration. (True - https://www.politifact.com/factchecks/2024/feb/08/kamala-harris/fact-checking-kamala-harris-did-the-biden-administ/)
5. An ostrich is a pink colored bird that stands on one leg. (False)
6. Galileo discovered gravity. (False)
7. Neil Armstrong was the first person on the moon. (True)
8. A one-lens eye piece is called a monocle. (True)
9. Joni Mitchell is dead. (False)
10. Dr. Seuss is alive. (False)
11. Stephen King is alive. (True)
12. Nina Simone is dead. (True)

13. Electric vehicles contribute fewer emissions that gasoline-powered cars over their lifetimes. (True - https://www.factcheck.org/2024/02/electric-vehicles-contribute-fewer-emissions-than-gasoline-powered-cars-over-their-lifetimes/)
14. Thermography is an effective and FDA approved alternative to mammograms. (False - https://www.factcheck.org/2023/07/scicheck-thermography-is-no-substitute-for-mammograms-contrary-to-facebook-posts-advice/)
15. There are no proven health risks for the general population from consuming the artificial sweetener aspartame. (True - https://www.factcheck.org/2023/10/no-proven-health-risks-from-aspartame-but-also-no-proven-benefits/)
16. Diagnoses of HIV in the U.S. military have increased 500% since the COVID-19 vaccine was mandated for service members (False - https://www.factcheck.org/2023/06/scicheck-database-errors-fuel-false-claims-about-hiv-cases-in-military/)
17. The assassination of John F. Kennedy was not committed by the lone gunman Lee Harvey Oswald but was rather a detailed organized conspiracy to kill the President. (Conspiracy – false)
18. The assassination of Martin Luther King Jr. was the result of an organized conspiracy by U.S. government agencies such as the CIA and FBI. (Conspiracy – false)
19. Princess Diana’s death was not an accident by rather an organized assassination by members of the British royal family who disliked her. (Conspiracy – false)
20. A powerful and secretive group known as the New World Order are planning to eventually rule the world though an autonomous world government which would replace sovereign governments. (Conspiracy – false)

Epistemic Beliefs Scales

Faith in Intuition for Facts Scale

1. I trust my gut to tell me what’s true and what’s not.
2. I trust my initial feelings about the facts.
3. My initial impressions are almost always right.
4. I can usually feel when a claim is true or false even when I can’t explain how I know.

Need for Evidence Scale

5. Evidence is more important than whether something feels true.
6. A hunch needs to be confirmed with data.
7. I trust the facts, not my instincts, to tell me what is true.
8. I need to be able to justify my beliefs with evidence.

Truth is Political Scale

9. Facts are dictated by those in power.
10. What counts as truth is defined by power.

11. Scientific conclusions are shaped by politics.
12. “Facts” depend on their political context.

Conspiracy Mentality Scale

1. … many very important things happen in the world, which the public is never informed about.
2. … politicians usually do not tell us the true motives for their decisions.
3. … government agencies closely monitor all citizens.
4. … events which superficially seem to lack a connection are often the result of secret activities.
5. … there are secret organizations that greatly influence political decisions.

Political Ideology

What is your political ideology?

1. Very liberal
2. Liberal
3. Somewhat liberal
4. Moderate
5. Somewhat conservative
6. Conservative
7. Very conservative

SUPPLEMENTARY TABLES AND FIGURE

SUPPLEMENTARY TABLE S1. *CORRELATION MATRIX*

Correlations

	1	2	3	4	5	6	7	8	9	10	11
1) Processing Fluency Condition	--										
2) Task Condition	0.009	--									
	.773										
3) Processing Fluency Scale	0.369**	0.124**	--								
	.000	.000									
4) Internal Efficacy Scale	-.066*	0.025	0.164**	--							
	.036	*.424*	*.000*								
5) Accuracy (*D'*)	-0.010	-0.040	0.065*	0.068*	--						
	.749	*.209*	*.039*	*.032*							
6) Bias [ln(β)]	0.022	0.016	-0.081*	-0.117**	-0.606**	--					
	.490	*.605*	*.010*	*.000*	*.000*						
7) Ideology	0.009	0.044	-0.053	0.086**	-0.249**	0.075*	--				
	.787	*.162*	*.096*	*.006*	*.000*	*.018*					
8) Conspiracy Mentality Scale	-0.032	-0.004	-0.074*	0.057	-0.353**	0.112**	0.271**	--			
	.306	*.890*	*.019*	*.073*	*.000*	*.000*	*.000*				
9) Faith in Intuition for Facts Scale	-0.010	-0.016	-0.041	0.140**	-0.290**	0.102**	0.206**	0.377**	--		
	.752	*.611*	*.200*	*.000*	*.000*	*.001*	*.000*	*.000*			
10) Need for Evidence Scale	-0.054	-0.003	0.114**	0.141**	0.334**	-0.161**	-0.272**	-0.225**	-0.360**	--	
	.086	*.912*	*.000*	*.000*	*.000*	*.000*	*.000*	*.000*	*.000*		
11) Truth is Political Scale	0.013	0.018	-0.086**	0.071*	-0.305**	0.101**	0.281**	0.508**	0.380**	-0.307**	--
	.690	*.579*	*.006*	*.025*	*.000*	*.001*	*.000*	*.000*	*.000*	*.000*	

Notes: The Pearson correlation is shown in the first row, with the *p* value in the second row of each variable. **. Correlation is significant at the 0.01 level (2-tailed), *. Correlation is significant at the 0.05 level (2-tailed).
